# Mental Health, Religion, and Spirituality: Knowledge, Attitude, and Practice Among Psychiatrists and Religious Leaders in Baguio City, Philippines

**DOI:** 10.1192/bjo.2024.275

**Published:** 2024-08-01

**Authors:** Cristina Agustin

**Affiliations:** Baguio General Hospital and Medical Center, Baguio, Philippines. University of the Philippines-Philippine General Hospital, Manila, Philippines

## Abstract

**Aims:**

To assess the knowledge, attitude and practices of psychiatrists and religious leaders in Baguio City, Philippines regarding mental health, religion, and spirituality.

**Methods:**

Two sets of questionnaires were adapted from the study of Foskett et al. (2004). Some questions and choices for the corresponding choices for answer were modified according to the objectives of this project. The questionnaires were prepared to collect data on knowledge, attitude and practices of psychiatrists and religious leaders regarding mental health, religion/spirituality (R/S). The questionnaire covered three main areas: (1) the links between mental health, R/S, (2) the state of collaboration between psychiatrists and religious leaders, and (3) the training each had received that was relevant to this area of their work. The platform used in the Data Collection is via Google Forms. In this method, identity of the responders were anonymized. Data like the name, clinic or office address, age, sex, religious affiliation of the responders were not collected. Descriptions and interpretations of the results were done using frequency and percentages and histograms.

**Results:**

Among the psychiatrists in Baguio City, only 58.8% responded to the questionnaire. Percentage of R/S leaders who responded could not be accounted due to insufficient data on the registry of all religions/spiritual groups in Baguio.

Neither disciplines has any doubts that there is a link between mental health, and R/S.

Majority of the psychiatrist respondents recognize the relevance of their own religion and spirituality. Their belief and inner resources were integral for their coping and implicitly affects their work.

Although majority of the psychiatrists think that referring a patient to R/S leaders should always be the case, and referring the terminally ill will be useful.

Majority (80%) of the psychiatrist respondents are not familiar with their institution's chaplain coordinator/unit. Also, majority (80%) never made a referral to the chaplain. It is also noted that 50% of the psychiatrist respondents did not receive referral from R/S leaders.

Majority of psychiatrists responders did not receive training on R/S aspect of mental health prior and during their qualification.

On the other hand, about 35% of the R/S leaders have a training on mental health prior and during their qualification. Religious/spiritual leader respondents equally think that they need further training on mental health.

**Conclusion:**

Psychiatrist and religious/spiritual leaders both recognize the role of R/S in mental health. The relationship of the two professions in collaborating still needs strengthening by collaboration, education, and training.

